# Impact of nasal septal perforation on the airflow and air-conditioning characteristics of the nasal cavity

**DOI:** 10.1038/s41598-024-52755-4

**Published:** 2024-01-29

**Authors:** Yang Na, Kyung Won Kwon, Yong Ju Jang

**Affiliations:** 1 Department of Mechanical Engineering, Konkuk University, Seoul, 05029 Korea; 2 Department of Otolaryngology, Asan Medical Center, University of Ulsan, College of Medicine, 88 Olympic-ro 43-gil, Songpa-gu, Seoul, 05505 Korea

**Keywords:** Computational science, Computational models, Experimental models of disease

## Abstract

We investigated (1) how nasal septal perforations (NSPs) modify nasal airflow and air-conditioning characteristics and (2) how the modifications of nasal airflow are influenced by the size and location of the NSP. Computed tomography scans of 14 subjects with NSPs were used to generate nasal cavity models. Virtual repair of NSPs was conducted to examine the sole effect of NSPs on airflow. The computational fluid dynamics technique was used to assess geometric and airflow parameters around the NSPs and in the nasopharynx. The net crossover airflow rate, the increased wall shear stress (WSS) and the surface water–vapor flux on the posterior surface of the NSPs were not correlated with the size of the perforation. After the virtual closure of the NSPs, the levels in relative humidity (RH), air temperature (AT) and nasal resistance did not improve significantly both in the choanae and nasopharynx. A geometric parameter associated with turbinate volume, the surface area-to-volume ratio (SAVR), was shown to be an important factor in the determination of the RH and AT, even in the presence of NSPs. The levels of RH and AT in the choanae and nasopharynx were more influenced by SAVR than the size and location of the NSPs.

## Introduction

Although approximately 40% of the patients with nasal septal perforations (NSPs) are asymptomatic^1^, common complaints in symptomatic cases include crusting, recurrent epistaxis, nasal obstruction, pain, nasal dryness, and nasal whistling^2–4^. In particular, most typical clinical symptoms such as local crust formation and bleeding observed at the posterior surface of NSPs are attributable to local crossover airflow through the perforations^5–14^ and, therefore, the size and location of the perforation can be important determinants of local flow disturbance.

Computational fluid dynamics (CFD) has become an effective analysis tool for the investigation of airflow and air-conditioning characteristics in the nasal cavity^15–21^ and this technique has been extended to study the effects of NSPs on nasal airflow in virtually created^6–9,14,22,23^ or true NSP models^10–13,24–26^. Due to the challenges associated velocity measurement in the highly deteriorated anatomy of the nasal cavity, especially in the presence of NSP, direct in vivo measurement studies are scarce. The exception to this scarcity is the very early work focused on temperature measurement^27,28^.

Previous numerical studies involving virtually generated NSP models^7–9^ have revealed the adverse effects of anterior NSPs. Moreover, numerical investigations were conducted in nasal cavity models with true NSPs^10–13,24–26^ and they confirmed the occurrence of increased WSS around perforations. Although the dependence of crossover airflow on perforation size has been discussed in some studies^12,25^, the impact of perforation size has not been sufficiently examined with real NSP models because of the limited number of samples^10,11,13,24,26^.

In addition to local airflow disruption, there are conflicting reports on the exact effect of NSPs on the deterioration of humidification and heating of inspired air in the nasopharynx^22,23,25,26^. These inconsistent observations raise questions about the precise impact of NSPs on the overall air-conditioning capacity of the nasal cavity.

Considering that NSPs are often iatrogenic after previous nasal surgery^3,29–31^, both nasal airflow and air-conditioning characteristics of nasal cavities with NSPs are likely to be strongly influenced by the structural features of the cavity resulting from the previous surgery. Therefore, the airflow characteristics in the presence of real NSPs are likely to differ from those of virtual NSPs created in the healthy cavities without any pathological problems. In this context, more realistic investigations with actual NSPs are necessary to better understand nasal airflow disturbed by NSPs.

The main objective of this study was to investigate, using CFD, the characteristics of local airflow around the perforations and the overall air-conditioning capacity of the nasal cavities with actual NSPs. The effects of the size and location of the NSPs on the airflow parameters were also examined.

## Materials and methods

### Patients

NSP was assessed by two otolaryngologists by nasal endoscopic examination, supplemented by routine diagnostic computed tomography (CT) image analysis. Patients with ongoing chronic rhinosinusitis or a history of sinus surgery, nasal polyposis, or neoplastic conditions were excluded. After evaluation, 14 patients with confirmed NSP (denoted as SP1-SP14) were selected.

### Ethics approval and consent to participate

This study was approved by the ethics committee of the Asan Medical Center (ethics number: 2020-0118). As the patient information was anonymized and deidentified before analysis, the ethics committee of the Asan Medical Center waived the need for informed consent. All methods were performed following the Declaration of Helsinki.

### Construction of nasal cavity models

**Figure 1:**
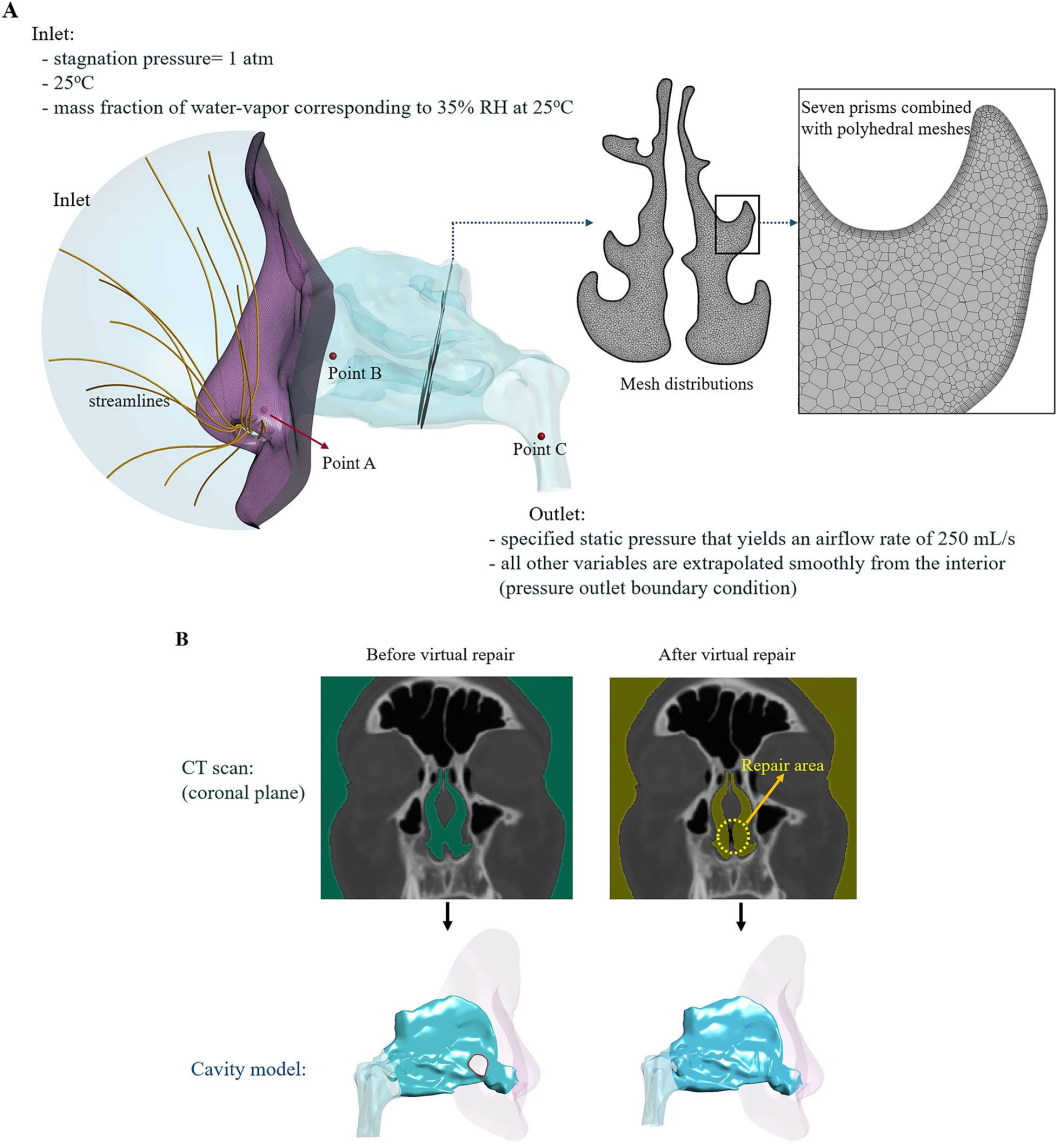
Schematic illustration of the numerical cavity model for the representative subject SP1. (**a**) Numerical cavity model with grid distribution. (**b**) Virtual repair procedure utilizing CT scans and the resultant nasal cavity models.

Nasal cavity models were constructed using osteo-meatal unit CT scans of the study subjects. The segmentation procedure was performed using Mimics v23.0 (Materialise, Leuven, Belgium), in which external features of the face of the subjects were included in the nasal cavity models to obtain more realistic airflow through the nostrils^17,32,33^. Figure 1A depicts the numerical cavity model for the representative subject (SP1). Virtual repair of NSPs was performed using CT scan images and Mimics v23.0. The perforations were closed manually so that the artificially repaired septal wall was consistent with the adjacent curvature as shown in Fig. 1B. The paranasal sinuses were removed from the cavity models, as in previous studies^6,8,9,14,25,33–35^.

### Classification of NSP by size and location

**Figure 2:**
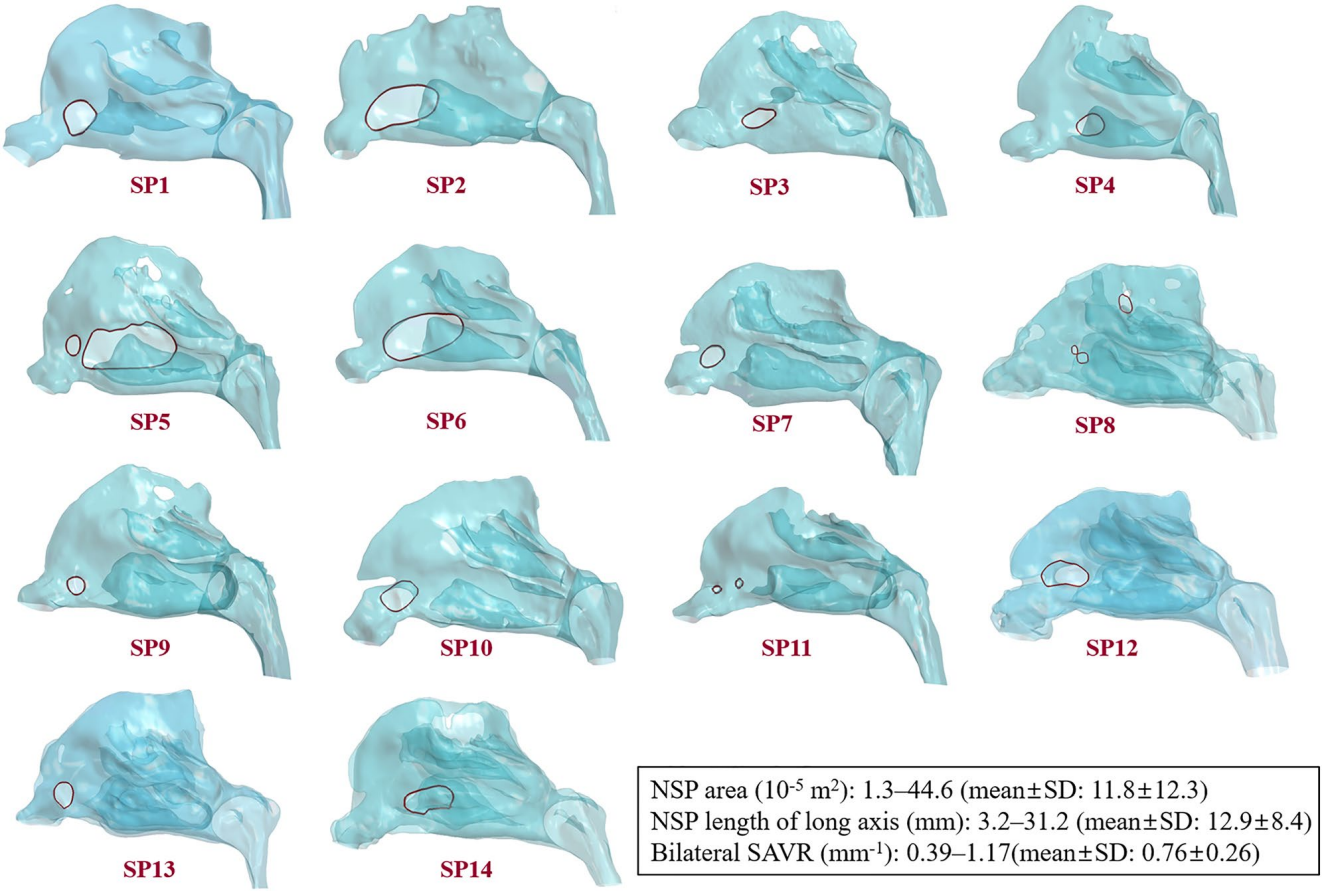
Nasal cavity models of the present study group (SP1–SP14). Nasal septal perforations are indicated by red lines. For visual convenience, the faces of the subjects were excluded.

The nasal cavities of the study group (SP1-SP14) and their NSPs are shown in Fig. 2. There are different criteria for the location of the NSPs in the literature^1,8,9^; we adopted the method of Cannon et al.^8^, in which the head of the inferior turbinate was used to define the cutoff location of the anterior and posterior perforations. Consequently, SP1, SP7, SP9, SP10 and SP13 were classified as anterior NSPs and SP3, SP4, and SP14 were classified as posterior NSPs. On the contrary, three subjects (SP2, SP5 and SP6) had relatively large NSP extending from the anterior to mid-cavity and were classified as mixed NSP. The remaining cavities (SP8, SP11, and SP12) did not belong to the subgroups mentioned above.

### CFD methodology

Airflow fields in the nasal cavity were simulated using ANSYS/Fluent 2021R2 software (Canonsburg, PA, USA) with a second-order central differencing scheme. The air temperature (AT) and relative humidity (RH) values were obtained by solving the energy and species transport equations for air and water vapor mixtures. For the evaluation of epithelial surface temperatures and the mass fraction of water vapor required in the energy and species transport equations, the wall model described in our previous studies^21,35^ was used, with an assumption of 100% RH along the epithelial surface. Validation of the numerical methodology was conducted in those prior studies^21,35^ using the velocity and temperature measurement data available in the literature.

Ambient conditions, prescribed at the inlet of the domain shown in Fig. 1A, were assumed to be a stagnation pressure of 1 atmospheric pressure, 25 °C, and 35% RH. Therefore, the mass fraction of water vapor was calculated such that 35% RH was achieved at 25 °C. The pressure prescribed at the outlet of each computational model was adjusted to achieve a constant inspiratory flow rate of 250 mL/s, which is the average airflow rate during inspiration from calm breathing, and a laminar flow regime was assumed, as in previous studies^17,19,21,36–38^. All variables except static pressure at the outlet were extrapolated from the interior smoothly according to the pressure boundary condition of ANSYS/Fluent R21.2.

Mesh elements, which combine seven prism layers with a growth rate of 1.15 along the surface (with the first layer thickness of 0.02 mm) and polyhedral elements away from the surface, were generated using Fluent Meshing 2021R2, as shown in Fig. 1A. A grid independence study was conducted for the representative subject (SP1) with 1.2, 2.2, 4.3, 6.5, and 8.6 million mesh elements. Variations in air velocity, temperature, and mass fraction of water vapor were examined at three representative locations shown in Fig. 1A. The results summarized in Table 1 indicate that 6.5 million elements were appropriate for resolving the flow fields.

**Table 1:** Variations in air velocity, temperature, and mass fraction of water vapor with the number of mesh elements at three representative locations depicted in Fig. 1A.

Location	Variables	Number of mesh elements				
		1.2 M	2.2 M	4.3 M	6.5 M	8.6 M
Point A (in vestibule)	Velocity (m/s)	2.263	2.263	2.259	2.255	2.254
	Temperature (°C)	25.02	25.01	25.00	25.00	25.00
	Mass fraction, water vapor (10^–2^)	0.6914	0.6921	0.6924	0.6925	0.6926
Point B (in middle meatus)	Velocity (m/s)	2.580	2.720	2.784	2.807	2.808
	Temperature (°C)	25.83	25.52	25.30	25.21	25.20
	Mass fraction, water vapor (10^–2^)	0.7589	0.7177	0.6874	0.6836	0.6833
Point C (in nasopharynx)	Velocity (m/s)	2.302	2.233	2.458	2.466	2.469
	Temperature (°C)	31.17	31.23	30.39	30.08	30.05
	Mass fraction, water vapor (10^–2^)	0.2342	0.2422	0.2077	0.1961	0.1957

Bilateral nasal resistance (NR) was estimated using the pressure difference between the inlet and choanae. The geometric parameter, the surface area-to-volume ratio (SAVR), was evaluated by calculating the surface area and the volume of the airway from the nostrils to the end of the septum.

### Statistical analyses

Pearson’s correlation coefficients (*r*-value) were used to assess the statistical correlations between the CFD variables. Student's two-tailed paired *t* tests were used to assess statistically significant differences, with *p*-value less than 0.05.

## Results

The average age of the entire cohort was 43.4 years and this population consisted of 13 men. NSPs were caused by previous septoplasty in 12 patients and the causes of NSPs were unclear in the remaining two patients. The NOSE-Perf^39^ score, a clinical assessment tool for patient-reported NSP symptoms, including nasal obstruction, dryness, nasal whistling, bleeding, facial pain, smell, and rhinorrhea, was 15.0 ± 6.9. The most frequent patient-reported symptom was nasal crusting/dryness (79%), followed by nasal obstruction (71%), epistaxis (29%), and whistling (21%).

### Geometric characteristics of the septal perforations

The geometric characteristics of the NSPs and the nasal cavities are summarized in Fig. 2. The median diameter of NSPs was estimated to be 1.5 cm^28^ and an NSP diameter greater than 2 cm was considered large in multiple studies^12,40^. Therefore, the following size criterion based on the length of the long axis was used: small (< 1 cm), medium (1 to 2 cm) and large NSP (> 2 cm). Among the anterior NSPs, three subjects (SP7, SP9, and SP13) had small perforations and two subjects (SP1 and SP10) had medium-sized perforations. All subjects with posterior NSPs (SP3, SP4, and SP14) had medium-sized perforations, while three subjects with mixed NSPs (SP2, SP5, and SP6) had large perforations. Perforation areas ranged from 1.3 $\times 1{0}^{-5}$ (SP11) to 44.6 $\times 1{0}^{-5}\,{{\text{m}}}^{2}$ (SP5). The characteristic size of each NSP, expressed by the length of its long axis, is in the range of 3.2 to 31.2 mm. The bilateral SAVR of the nasal cavity ranged from 0.39 to 1.17 mm^−1^.

### Local effects on airflow around the septal perforations

**Figure 3:**
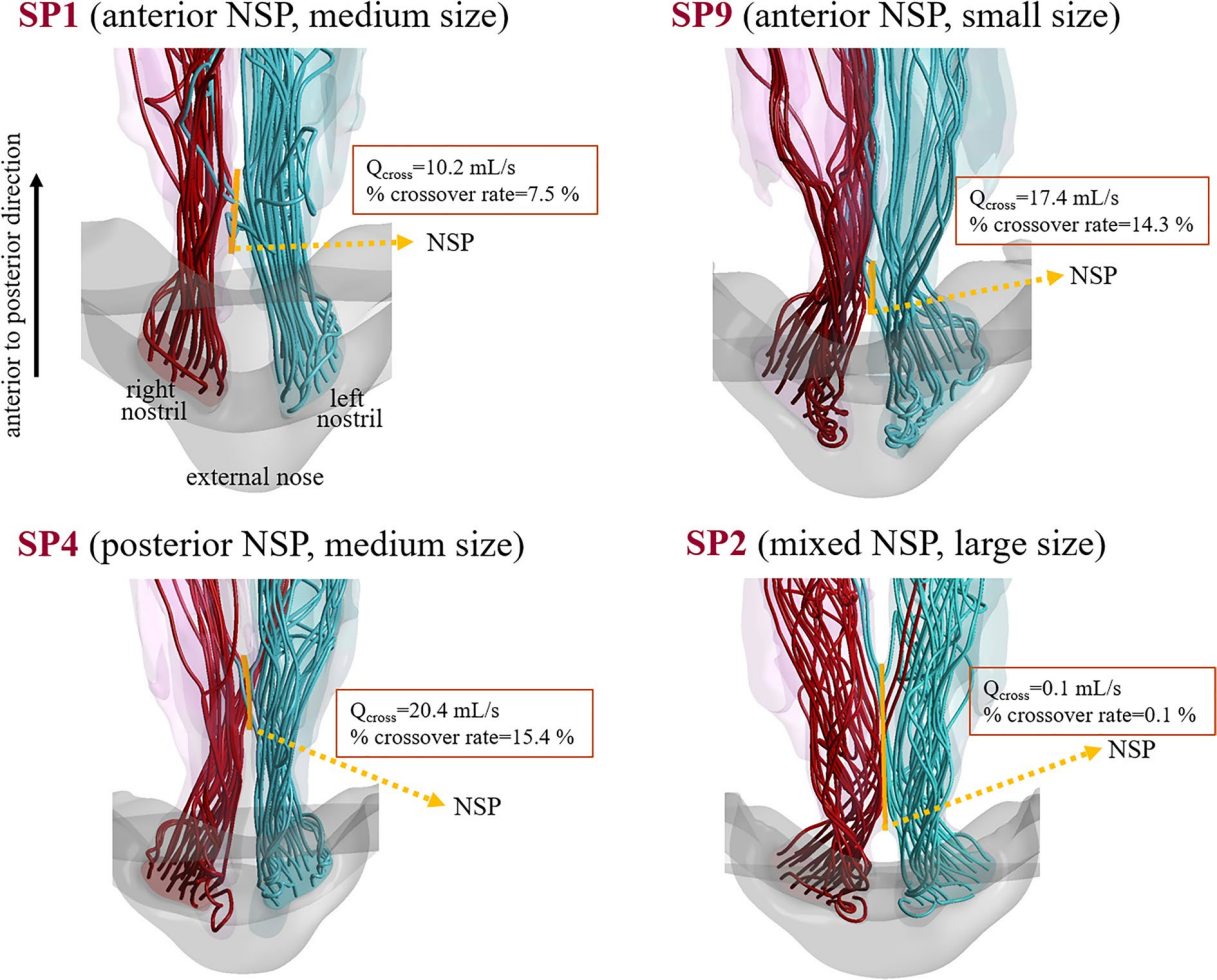
Axial view of streamline patterns for SP1, SP9, SP4, and SP2. SP1 and SP9 represent nasal cavities with anterior NSPs while SP4 and SP2 represent cavities with a posterior and a mixed NSPs.

The streamline patterns for four representative subjects (SP1, SP9, SP4, and SP2) are shown in Fig. 3. SP1 and SP9 represent cavities with medium (1–2 cm) and small (< 1 cm) anterior NSPs, respectively. SP4 represents the cavity with a medium posterior NSP, whereas SP2 represents the cavity with a large mixed NSP (> 2 cm). It was noted that the net crossover airflow rate and the percentage crossover airflow rate, that is, the ratio of the net crossover airflow rate to the unilateral airflow rate of the cavity side from which the crossover originates, varied significantly irrespective of the size of the perforation. In particular, SP2 with a mixed NSP, which had the largest NSP area among the four subjects, produced the smallest net crossover airflow rate (see Supplementary Data for individual data).

The CFD variables related to the local airflow around the perforations for all study subjects (SP1–SP14) are summarized in Table 2. Because the airflow can cross through the perforation from both side of the airway, the amount of airflow originating from each side was evaluated separately before calculating the net crossover airflow rates. Net airflow rates through the perforation varied substantially (0.1–57.7 mL/s) and were not correlated with the NSP area (*r* = 0.026, *p* = 0.303). Significantly increased WSS and surface-water vapor flux were observed on the posterior surface of the NSP. However, these two parameters were not correlated with the area of the NSP (*r* = −0.101, *p* = 0.732 and *r* = −0.098 and *p* = 0.739, respectively) or with the net airflow rate through the NSP (*r* = 0.157, *p* = 0.595 and *r* = −0.0238 and *p* = 0.936, respectively).

**Table 2:** CFD variables in relation to nasal septal perforations in all cavity models of the study group.

CFD variables	Range	Mean ± SD	Correlation with NSP area	Correlation with net airflow rate through the NSP
Net airflow rate through NSP (mL/s)	0.1–57.7	18.2 ± 17.4	0.026 (*p* = 0.930)	1.000
Max. velocity in the cross section of NSP (m/s)	1.31–3.98	2.82 ± 0.70	0.277 (*p* = 0.338)	0.4580 (*p* = 0.100)
Max. wall shear stress on posterior surface of NSP (Pa)	0.42–1.38	0.80 ± 0.30	−0.101 (*p* = 0.732)	0.157 (*p* = 0.595)
Max. surface water–vapor flux on posterior surface of NSP (10^–2^ kg/s/m^2^)	0.29–0.60	0.0043 ± 0.0009	−0.098 (*p* = 0.739)	−0.0238 (*p* = 0.936)

*NSP* nasal septal perforation.

Correlation was estimated using Pearson’s correlation coefficient (*r*-value).

The information shown in Table 2 was further broken down into subgroups classified by NSP location (Table 3). Net airflow rate and maximum velocity through the perforation of the anterior NSPs were found to be lower than those of the posterior NSPs (13.9 vs. 27.0 mL/s and 2.64 vs. 3.12 m/s, respectively). However, the WSS on the posterior surface of the perforation was somewhat higher in the anterior NSPs than in the posterior NSPs (0.88 vs 0.76 Pa). In particular, medium-sized anterior NSPs exhibited higher values in the velocity through the perforation, as well as in the WSS and surface water–vapor flux than small-sized anterior NSPs despite the lower net airflow rate through the NSPs.

**Table 3:** CFD variables in relation to the nasal septal perforations in cavity models with anterior, posterior, and mixed NSPs.

CFD variables	Anterior NSP (SP1, 7, 9, 10, 13)		Posterior NSP (SP3, 4, 14)	Mixed NSP (SP2, 5, 6)
	Small NSP (SP7, 9, 13)	Medium NSP (SP1, 10)		
Net airflow rate through NSP (mL/s)	13.9 ± 9.0		27.0 ± 28.0	12.0 ± 10.6
	15.2 ± 12.4	12.0 ± 2.7		
Max. velocity in the cross section of NSP (m/s)	2.64 ± 0.51		3.12 ± 0.61	3.09 ± 0.79
	2.42 ± 0.15	2.98 ± 0.81		
Max. wall shear stress on posterior surface of NSP (Pa)	0.88 ± 0.36		0.76 ± 0.21	0.77 ± 0.44
	0.73 ± 0.32	1.11 ± 0.39		
Max. surface water–vapor flux on posterior surface of NSP (10^–2^ kg/s/m^2^)	0.43 ± 0.09		0.43 ± 0.01	0.42 ± 0.09
	0.40 ± 0.11	0.47 ± 0.00		

*NSP* nasal septal perforation.

Correlation was estimated using Pearson’s correlation coefficient (*r*-value).

### Effect of NSP on NR, RH, AT in the nasopharynx

The bilateral NR, RH, and AT measured in the nasopharynx and their correlations with the size of the NSP and SAVR are summarized in Table 4. Bilateral NR showed significant variations, but no correlation was evident with the NSP area (*r* = −0.214, *p* = 0.463). However, bilateral NR exhibited a high correlation with bilateral SAVR (*r* = 0.830, *p* < 0.001). Similarly, RH and AT in the nasopharynx showed considerable variation, but were not correlated with the NSP area (*r* = −0.289, *p* = 0.317 and *r* = −0.373, *p* = 0.189, respectively). Instead, they exhibited a strong correlation with bilateral SAVR (*r* = 0.880 and 0.861 with *p* < 0.001 for RH and AT, respectively).

**Table 4:** Bilateral nasal resistance, relative humidity, and air temperature values measured in the nasopharynx before virtual closure of NSPs.

CFD variables	Range	Mean ± SD	Correlation with NSP area	Correlation with bilateral SAVR
Bilateral nasal resistance (Pa/mL/s)	0.0173–0.0652	0.0350 ± 0.0143	−0.214 (*p* = 0.463)	0.830 (*p* < 0.001)
Relative humidity (%)	76.9–97.4	87.6 ± 6.6	−0.289 (*p* = 0.317)	0.880 (*p* < 0.001)
Air temperature (°C)	30.4–32.5	31.4 ± 0.7	−0.373 (*p* = 0.189)	0.861 (*p* < 0.001)

*NSP* nasal septal perforation, *SAVR* surface area-to-volume ratio.

Correlation was estimated using Pearson’s correlation coefficient (*r*-value).

### Effect of virtual closure of NSPs on flow partition, NR, RH and AT

Table 5 summarizes the flow partition and unilateral NR ratios, and the differences in RH and AT between the left and right cavities measured in the choanae before and after the virtual closure of the perforations for all study subjects. It should be noted that the repair of NSPs did not significantly alleviate differences in RH and AT, as well as flow partition and unilateral NR ratios in the choanae on average.

**Table 5:** Ratios of flow partition and unilateral nasal resistance in the choanae, differences between the two sides of the cavity in relative humidity and air temperature evaluated in the choanae, bilateral nasal resistance, relative humidity, and air temperature measured in the nasopharynx before and after virtual closure of the perforations.

CFD variables		Range	Mean ± SD
Ratio of flow partition in the choanae (larger side/lower side)	Before closure	1.02–2.15	1.36 ± 0.34
	After closure	1.07–2.02	1.36 ± 0.48
Ratio of unilateral NR in the choanae (larger side/lower side)	Before closure	1.02–4.04	1.40 ± 0.78
	After closure	1.03–2.78	1.37 ± 0.49
Difference in relative humidity between two cavity sides in the choanae (%)	Before closure	0.7–9.8	4.8 ± 2.9
	After closure	0.5–9.8	4.9 ± 2.6
Difference in air temperature between two cavity sides in the choanae (°C)	Before closure	0.1–1.1	0.6 ± 0.3
	After closure	0.1–1.1	0.6 ± 0.3
Bilateral nasal resistance (Pa/mL/s)	Before closure	0.0173–0.0652	0.0350 ± 0.0143
	After closure	0.0175–0.0661	0.0358 ± 0.0149
	*p*-value	0.081	-
Relative humidity (%)	Before closure	76.9–97.4	87.6 ± 6.6
	After closure	76.6–97.5	88.0 ± 6.1
	*p*-value	0.379	
Air temperature (°C)	Before closure	30.4–32.5	31.4 ± 0.7
	After closure	30.5–32.5	31.4 ± 0.6
	*p*-value	0.195	-

*NR* nasal resistance.

Modification of the CFD variables in the nasopharynx after virtual closure of the NSPs is summarized in Table 5. Bilateral NR increased slightly after repair, but this change was not considered significant (*p* = 0.081). Similarly, the changes in RH and AT in the nasopharynx were not significant after repair (*p* = 0.379 and 0.195, respectively) (see Supplementary Data for individual data).

## Discussion

We have confirmed the results of previous studies that the air exchange through the perforations disrupts the local airflow and causes an increase in WSS on the posterior margin of the perforations^5–14^. Furthermore, the surface water–vapor flux on the posterior surface was found to increase substantially. This finding is also in line with the results of a study with artificially created anterior NSPs^14^. Because an elevated water vapor flux from the epithelial surface to inhaled air, in turn, would lead to a possible dehydration of the mucosal surface, the excessive loss of mucus from the surface is likely to be the basis for crust formation and epistaxis^2,41^.

The distinctive result of the present study is the observation that several parameters, such as the net crossover airflow rate, the velocity through the perforation, and the level of WSS on the posterior surface of the perforations, were not correlated with the size of the NSP. The lack of association between NSP size and crossover airflow rate in our study is not in agreement with the findings of several studies that have used artificially generated NSP models. For example, earlier studies have indicated that flow disturbance due to NSPs is dependent on the size of the perforation^7,9^. If restricted to the cavities of the anterior NSPs, the agreement between the present results and those of prior studies with virtual anterior NSPs^8,14^ is somewhat mixed. That is, agreement is achieved in the observation that the velocity and WSS on the posterior surface of the perforation increased with the size of the NSP suggesting that anterior NSP may be more symptomatic than posterior perforations, whereas our findings on the dependence of the net airflow rate and water vapor flux on the size of the NSP are in discordance with their results. These discrepant results may be due to the fact that previous studies, unlike our current analyses, used a single healthy subject without pathology and virtually created NSPs that did not have any deteriorated anatomy as a baseline cavity model. We expect that these systems may not accurately reflect real NSP characteristics, as they are frequently associated with a surgically altered nasal cavity structure, such as reduced turbinate volume. Because the crossover airflow through the perforations is determined by the local anatomical geometry and associated flow dynamics, a simple relationship was not likely to be found between the NSP size and the crossover airflow rate (as well as the levels of WSS or surface water–vapor flux). Our present analyses confirmed that the correlations between the size and the net airflow through the perforations were small. It is noteworthy that SP2, who had a mixed NSP and the largest NSP area among the four subjects shown in Fig. 3, produced almost identical crossover airflow from each side of the NSP, resulting in a very small net crossover airflow rate.

Differences in intranasal RH or temperature fields between the left and right nasal cavities were also investigated. The substantial difference between the left and right sides reported by Pless et al.^22^ was not observed in Lindemann et al.^23,42^. Our results indicated that the RH difference between the two sides of the cavity assessed at the location of the choanae varied widely between subjects before closure of NSPs (0.7 to 9.8%, Table 5). Interestingly, the largest difference of 9.8% (SP6) and the smallest difference of 0.7% (SP2) were found in cavities with mixed NSPs with large perforations (> 2 cm). Therefore, we can infer that the RH asymmetry between the two sides of the cavity is unlikely to be highly correlated with the NSP size. Furthermore, the fact that differences in RH between the two sides of the cavity were not noticeably alleviated after virtual closure (Table 5) suggests a limited physiological role of NSPs on the level of RH in the choanae.

The importance of the geometric characteristics of the nasal cavity, not the size of the NSP, in the overall air-conditioning capacity^16,35^ was also confirmed in the presence of NSPs in terms of the strong correlation we observed between the RH and bilateral SAVR (Table 4). This result suggests that one of typical symptoms of NSP, such as nasal dryness throughout the airways resulting from impaired air-conditioning, is not directly related to the presence of NSP, but is more related to structural damage, including reduced turbinate volume, leading to low SAVR, caused by previous surgery. However, this result is not in agreement with the findings of a previous report by Li et al.^25^ with real NSPs, in which the impairment of the air-warming function was shown to be directly dependent on the size of the perforation. Further studies are needed to accurately assess this discrepancy in relation to the impact of NSP on the overall air-conditioning capacity of the nasal cavity.

Surgical closure of NSPs would obviously reduce undesirable stimulation of the epithelium on the posterior surface of this defect by eliminating local regions exposed to high levels of WSS and surface water–vapor flux. In this context, NSP repair can significantly reduce the symptoms that arise around the NSPs. However, the fact that virtual NSP closure did not induce noticeable changes in nasopharynx RH and AT (Table 5) suggests that this correction alone would not restore the impaired air-conditioning capacity of the nasal cavity. In our numerical experiments, the geometric parameter for the nasal cavity, SAVR, which decreases as the volume of the turbinate decreases, was shown to be more influential than the size and location of the NSPs in determining RH and AT in the nasopharynx. Hence, a comorbid condition involving iatrogenic turbinate reduction surgery can have a more adverse influence on the air-conditioning capacity of the nasal cavity than the presence of perforations.

The present study has several limitations. First, although a significantly larger number of samples were considered in our current analysis than in any previous study, the sample size still needs to be increased to reduce statistical fluctuations. In addition, our study design did not attempt to analyze any correlation between objective variables and subjective symptoms in patients with NSP, such as the NOSE-Perf score. As the virtual perforation repair cannot precisely simulate an actual repair, it would be beneficial to conduct a future study that correlates the patient-reported symptoms with the outcomes of both actual and virtual repairs. This would provide insights into the effectiveness of the virtual repair and its relevance to the patient’s experience. Since the present work investigated airflow characteristics at the average airflow rate of 250 mL/s during the inspiration period, prediction of the dynamic variation of airflow variables with the flow rate was not possible, and thus additional computations at different airflow rates would be useful to better understand the role of NSP during the whole inspiration period.

## Conclusion

The size of the NSP did not show strong correlations with the net cross-airflow rate through the perforations, the levels of WSS and surface water–vapor flux around the NSPs. However, for the anterior NSPs, the WSS and surface water–vapor flux were slightly higher for the larger NSPs. The levels of RH and AT in the choanae and nasopharynx were more influenced by SAVR than the size and location of the NSPs.

### Supplementary Information

Supplementary Information 1.

Supplementary Information 2.

Supplementary Information 3.

## Data Availability

All data generated or analysed during this study are included in this published article and its supplementary information files.
